# The Aryl Hydrocarbon Receptor in the Pathogenesis of Environmentally-Induced Squamous Cell Carcinomas of the Skin

**DOI:** 10.3389/fonc.2022.841721

**Published:** 2022-03-03

**Authors:** Christian Vogeley, Katharina M. Rolfes, Jean Krutmann, Thomas Haarmann-Stemmann

**Affiliations:** IUF - Leibniz-Research Institute for Environmental Medicine, Düsseldorf, Germany

**Keywords:** aryl hydrocarbon receptor (AHR), apoptosis, DNA repair, immunosuppression, ultraviolet radiation, skin cancer, polycyclic aromatic hydrocarbons

## Abstract

Cutaneous squamous cell carcinoma (SCC) is one of the most frequent malignancies in humans and academia as well as public authorities expect a further increase of its incidence in the next years. The major risk factor for the development of SCC of the general population is the repeated and unprotected exposure to ultraviolet (UV) radiation. Another important risk factor, in particular with regards to occupational settings, is the chronic exposure to polycyclic aromatic hydrocarbons (PAH) which are formed during incomplete combustion of organic material and thus can be found in coal tar, creosote, bitumen and related working materials. Importantly, both exposomal factors unleash their carcinogenic potential, at least to some extent, by activating the aryl hydrocarbon receptor (AHR). The AHR is a ligand-dependent transcription factor and key regulator in xenobiotic metabolism and immunity. The AHR is expressed in all cutaneous cell-types investigated so far and maintains skin integrity. We and others have reported that in response to a chronic exposure to environmental stressors, in particular UV radiation and PAHs, an activation of AHR and downstream signaling pathways critically contributes to the development of SCC. Here, we summarize the current knowledge about AHR’s role in skin carcinogenesis and focus on its impact on defense mechanisms, such as DNA repair, apoptosis and anti-tumor immune responses. In addition, we discuss the possible consequences of a simultaneous exposure to different AHR-stimulating environmental factors for the development of cutaneous SCC.

## Introduction

Non-melanoma skin cancers, in particular basal cell carcinoma and SCC, are among the most frequent malignancies in humans (1–3). Cutaneous SCC primarily develop on sun-exposed areas of the body. Accordingly, a chronic exposure to artificial (tanning beds) or solar UVB radiation and the associated accumulation of damaged keratinocytes is the most important risk factor for SCC (1–3). Due to the continuously growing number of elderly individuals in the general population as well as the unbroken popularity of tanned skin among younger generations, the incidence of SCC is predicted to further increase (1–3). This trend might be exacerbated by environmental, occupational and life style-related exposure to carcinogenic chemicals, especially combustion-derived PAHs, alone or in combination with UV exposure (3). In addition, the climate change and associated global weather shifts may have an impact on human health and will probably increase the incidence of skin cancers and other malignancies (4, 5). Because SCC is not only a growing medical problem but also a substantial economic burden to health care systems (3, 6), there is an urgent need for the development of novel preventive and therapeutic measures. In this context, the AHR, a ligand-activated transcription factor and key regulator in xenobiotic metabolism and immunity, seems to be a promising molecular target. This notion is strengthened by the outcome of a two-stage genome-wide association study identifying the AHR as a novel susceptibility locus for cutaneous SCC in humans (7).

In this review article, we focus on the critical functions of AHR for DNA damage-dependent processes and immune responses which may contribute to the development of SCC in chronically UV- and/or PAH-exposed skin. Please note that while we are focusing on the mentioned aspects other functions of the AHR system might fall short, which may be also relevant for the process of skin cancer development.

## AHR in Xenobiotic Metabolism and Chemical Carcinogenesis

The multistage model of carcinogenesis, defining the process of tumor development as a strict sequence of initiation, promotion and progression, was established more than 70 years ago (8, 9). In these studies, the researchers induced skin tumorigenesis in mice by applying 7,12-dimethylbenz[*a*]anthracene (DMBA) as tumor-initiator and phorbol ester-containing croton oil as tumor-promoting agent. Sequencing of the tumor DNA as well as further mechanistic studies provided evidence that PAHs initiate carcinogenesis by inducing mutations primarily in the *Ha-Ras* oncogene and that this process requires a metabolic conversion of the *per se* non-toxic chemicals to highly reactive metabolites, a process which is primarily carried out by AHR-regulated cytochrome P450 (CYP) monooxygenases (10–12).

### AHR Ligands and Signaling Pathways

The AHR belongs to the basic helix-loop-helix Per-ARNT-Sim superfamily of transcription factors whose members translate developmental, physiological and environmental signals into biochemical processes and cell biological responses (13). Within this protein superfamily, the AHR is the only member which is activated by binding of small molecular weight compounds (ligands). AHR ligands can be divided into exogenous and endogenous compounds (14–17). The list of exogenous AHR ligands encompasses environmental pollutants, such as PAHs and dioxins, plant- and microbiota-derived indoles and polyphenols, and pharmaceutical drugs. Indole derivatives, such as indolo[3,2-*b*]carbazole, 6-formylindolo[3,2-*b*]carbazole (FICZ) and 2-(1’H-Indole-3’-carbonyl)-thiazole-4-carboxylic acid methyl ester as well as tryptophan metabolites, such as kynurenic acid and xanthurenic acid, are considered as relevant endogenous agonists of AHR (14–17).

**Figure 1:**
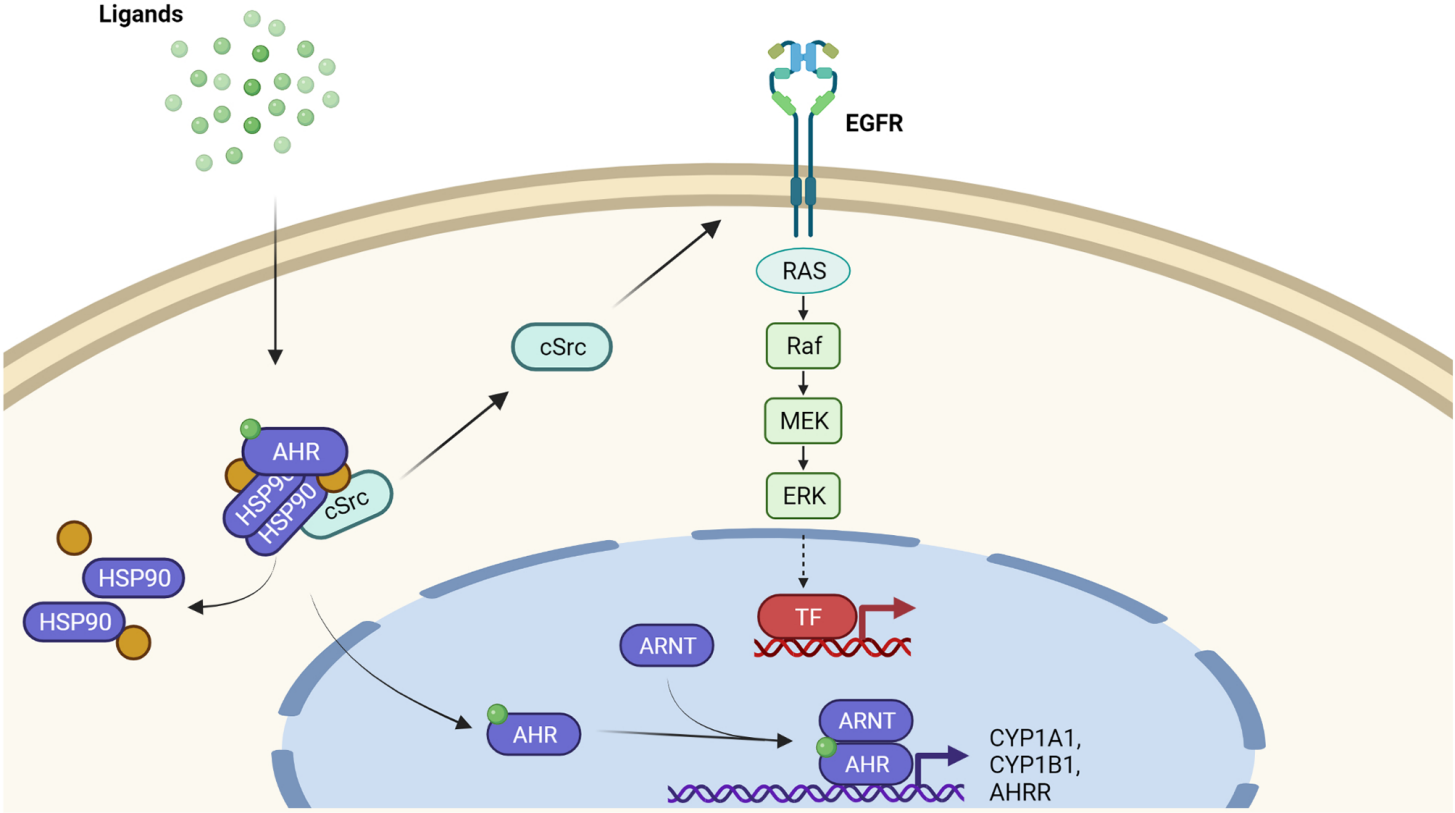
AHR-dependent signaling pathways. Inactive AHR is part of a cytosolic multiprotein. complex consisting of a HSP90 dimer, other chaperone molecules and tyrosine kinase c-Src. Upon ligand binding, the multiprotein complex dissociates and AHR translocates into the nucleus. Nuclear AHR heterodimerizes with ARNT and binds to the enhancer region of target genes, for instance encoding CYP1A1, CYP1B1, and AHRR, to enhance their expression. In addition to this canonical pathway, the AHR ligand-driven release of c-Src from the cytosolic multiportion complex may stimulate EGFR and downstream mitogen-activated protein kinases, resulting in the transcriptional induction of another set of genes.

In its inactive state, the AHR is trapped in a cytosolic multiprotein complex, composed of two heat-shock protein 90 (HSP90) molecules, the AHR-interacting protein, the co-chaperone p23 and the soluble tyrosine kinase c-Src (18) (**Figure 1**). Upon ligand binding, the cytosolic multiprotein complex dissociates, the AHR translocates into the nucleus, and dimerizes with its binding partner AHR nuclear translocator (ARNT). This heterodimer binds to xenobiotic-responsive elements (XRE) in the enhancer region of target genes to induce their expression (14–16). Typical AHR target genes encode for xenobiotic-metabolizing enzymes, such as CYP1A1, CYP1A2 and CYP1B1 (14–16). Another XRE-regulated gene codes for the AHR repressor, an AHR-related protein that lacks a transactivation domain and represses AHR signaling by competing for ARNT- and XRE-binding (19). Next to this so-called canonical AHR signaling pathway, the dissociation of the multiprotein complex leads to the release of c-Src, which subsequently may activate the epidermal growth factor receptor (EGFR) and downstream mitogen-activated protein kinase signal transduction (20–22) (**Figure 1**). Furthermore, AHR interacts with other transcription factors, including nuclear factor-κB (NF-κB) (23, 24), hypoxia-inducible factor-1α (25, 26), estrogen receptor-α (27, 28), and nuclear factor erythroid 2-related factor 2 (29, 30). These non-canonical functions may explain the frequently observed tissue- and cell-specific effects of AHR signaling and probably contribute to the pathogenesis of inflammatory and malignant diseases.

### AHR and Metabolic Activation of Polycyclic Aromatic Hydrocarbons

**Figure 2:**
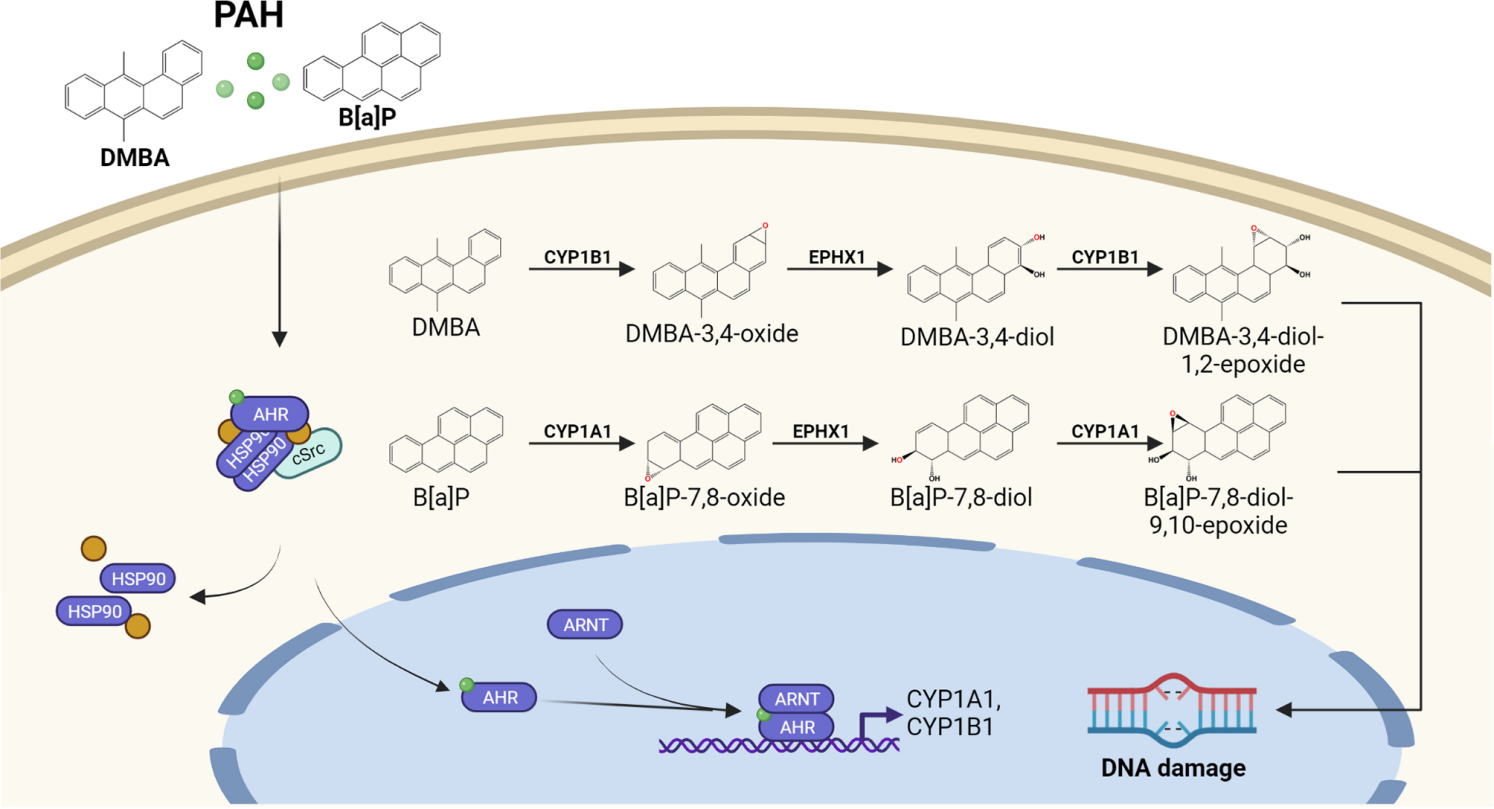
AHR-dependent metabolic activation of PAHs by CYP1 isoforms. Lipophilic PAHs can easily pass the plasma membrane and activate the canonical AHR signaling pathway. The resulting induction of CYP1 isoform expression and enzyme activity accelerates the oxidative metabolism of the invading chemicals. In case a proper detoxification of the resulting reactive PAH metabolites by the conjugating enzyme system (not shown) fails, reactive PAH-diol epoxides may covalently bind to the DNA and form highly mutagenic bulky DNA adducts.

Exposure to environmental, occupational and life-style related organic pollutants is considered to be involved in the onset of cutaneous SCC (3). Especially a long-lasting occupational exposure to PAHs present in soot and various working materials, such as coal tar, bitumen and petroleum, may facilitate skin carcinogenesis (3, 31). In addition, the elevated risk of smokers to develop cutaneous SCC is largely attributed to the PAH fraction present in tobacco smoke (32–34). The genotoxic potential of PAHs is primarily unleashed by their activation through AHR-dependent CYP1 isoforms (10–12) (**Figure 2**). For instance, CYP1A1 and microsomal epoxide hydrolase 1 (EPHX1) sequentially metabolize benzo[*a*]pyrene (B[a]P) to B[a]P-7,8-dihydrodiol-9,10-epoxide (BPDE), a highly carcinogenic compound which forms bulky DNA adducts by binding to guanine at the N2 position (10–12). Since CYP1A1 expression is regulated by the AHR, AHR-deficient mice as well as mice bearing an epidermis-specific ARNT-deletion were resistant towards B[a]P induced skin carcinogenesis (35, 36). In contrast to B[a]P, DMBA is metabolized by CYP1A1, CYP1B1 and EPHX1. Whereas CYP1A1 leads to the detoxification of DMBA, the CYP1B1-mediated oxidation results in an accumulation of highly carcinogenic DMBA-3,4-diol-1,2-epoxide (37, 38). CYP1-specific alterations in the detoxification and metabolic activation was also reported for other PAHs, such as dibenzo[*a,l*]pyrene (39) and dibenzo[*def,p*]chrysene (40). Thus, the carcinogenic potential of PAHs is determined by the CYP1 isoform predominantly expressed in the exposed cell population. Interestingly, the expression of CYP1B1 is not exclusively regulated by AHR (41–43) and, accordingly, AHR-deficient mice still express sufficient amounts of CYP1B1 to toxify DMBA and initiate skin carcinogenesis (44). Another carcinogenesis study revealed that AHR-deficiency protects mice against the skin carcinogenicity of PAH-rich airborne particulate matter (PM) (45).

At this point, we should mention that the CYP1-derived reactive PAH metabolites are efficiently detoxified through conjugation to glutathione, glucuronic acid or other hydrophilic molecules by phase 2 enzymes (11). However, in case the capacity of the conjugating enzyme system is exhausted, relevant amounts of reactive phase 1 metabolites may react with the DNA. Depending on the efficacy of other defense mechanisms, such as DNA repair and apoptosis, these DNA lesions may give rise to mutations (11, 46, 47).

The same is true for oxidative DNA damage that may occur during PAH metabolism. Specifically, CYP1-derived PAH dihydrodiols may serve as substrate for aldo-keto reductases (AKR), a family of cytosolic NADPH-dependent oxidoreductases (48). Several AKR1 isoforms, including AKR1C3, are capable of converting PAH dihydrodiols to the respective catechols which in the presence of oxygen can undergo redox-cycling. This results in the generation of reactive oxygen species (ROS), such as hydrogen peroxide and superoxide, which may contribute to skin carcinogenesis by oxidatively damaging DNA and other macromolecules (49, 50). In addition, AKR1C3 reduces prostaglandin (PG) D_2_ to 9α,11β-PGF_2_, a process which may fuel type 2 T helper (Th2) cell-related inflammatory responses in the skin (51, 52). Noteworthy, this AKR1C3-catalyzed reaction reduces the spontaneous dehydration of PGD_2_ to 15Δ-PGJ_2_, an eicosanoid that acts anti-inflammatory by inducing peroxisome proliferator-activated receptor-γ signaling and inhibiting pro-inflammatory NF-κB signaling pathways (51). Importantly, AKR1C3 is highly expressed in human SCC (53) and, moreover, we found that a PAH exposure of human keratinocytes results in an AHR-dependent upregulation of this enzyme (22). Taken together, these findings suggest that an AHR/AKR1C3-dependent modulation of PGD_2_ metabolism may foster the growth and apoptosis-resistance of initiated keratinocytes and SCC cells.

### AHR and PAH-Induced Immune Reactions

**Figure 3:**
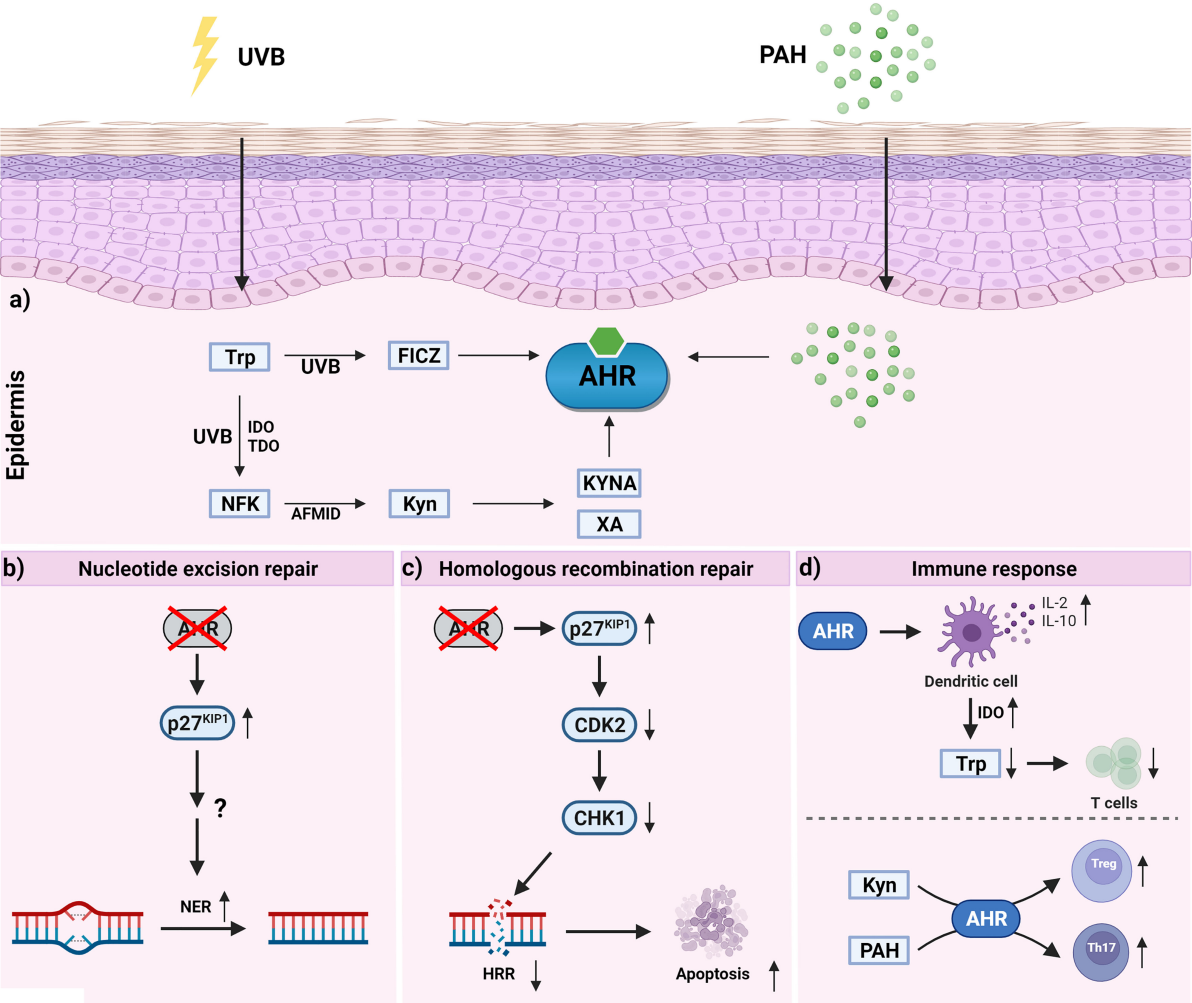
Activation of epidermal AHR signaling and AHR-dependent effects which may contribute to skin carcinogenesis. a) UVB irradiation of epidermal cells results in the formation of tryptophan photoproducts, such as FICZ and NFK. While FICZ is a high-affinity ligand for AHR, NFK is considered as a pro-ligand. Specifically, aryl formamidase converts NFK to kynurenine (kyn) which in subsequent metabolic reactions is metabolized to the AHR agonists kynurenic acid (KYNA) and xanthurenic acid (XA). b) Inhibition of AHR was shown to increase p27^KIP1^ protein content which improves NER of UVB radiation-induced DNA photoproducts by a yet enigmatic mechanism. c) Inhibition of AHR was shown to increase p27^KIP1^ protein content resulting in an inhibition of CDK2 and a time-dependent decline of the CHK1 level. The reduction of CHK1 is associated with a reduced performance of the HRR system and an elevated rate of keratinocyte apoptosis. d) AHR activation affects T cell polarization in a ligand-dependent manner. The upregulation of IL-2, IL-10 and IDO in DC may lead to an expansion of Tregs and inhibit the proliferation of effector T cells *via* tryptophan depletion. In contrast to the pro-ligand KYN, PAHs seem to interfere with differentiation and function of immunosuppressive Tregs.

An epicutaneous sensitization with PAHs, more precisely AHR/CYP1-derived reactive PAH metabolites, results in the development of an early inflammatory response which is responsible for contact hypersensitivity of the skin (54–56). This acute inflammation is triggered by antigen-specific CD8^+^ cytotoxic T cells and CD4^+^ type 1 T helper (Th1) cells which may prevent skin tumor development by secreting respective cytokines, such as interleukin (IL)-2 and interferon-γ (54–56). In its extent, this response is controlled by Th2 cells and immunosuppressive FoxP3^+^ regulatory T cells (Tregs). The induction of keratinocyte apoptosis by cytotoxic T cells leads to the release of further mediators which may stimulate other immune cells to infiltrate the inflamed skin. In case the damage is not resolved, chronic inflammatory condition facilitates the growth and progression of skin tumors (57). In experimental carcinogenesis studies, the application of phorbol ester fosters the expansion of IL-17-producing T cells which further promote tumor growth (58). Recently, various studies reported that by inhibiting the function of immunosuppressive Tregs (59–61) and promoting the polarization of Th2 and Th17 cells (**Figure 3**), an exposure to PAHs or PAH-rich particulate matter facilitates the worsening of chronic inflammatory diseases, including asthma and atopic dermatitis, in an AHR-dependent manner (61–67). Given that Th2 as well as Th17 responses are well recognized for their tumor-promoting capabilities (57), it is tempting to speculate that a chronic exposure of the skin to genotoxic PAH or PAH-rich materials may not only initiate the development of tumors but also promote their growth by creating an inflammatory micromilieu.

## Ultraviolet Radiation and Skin Photocarcinogenesis

Ultraviolet (UV) radiation is part of the electromagnetic spectrum of sunlight and can be subdivided into UVA, UVB and UVC radiation (68, 69). The latter (100 nm – 280 nm) is almost completely absorbed by the stratospheric ozone layer and thus does not reach our skin in sufficient amounts to cause biological effects. High-energy UVB photons (280 nm – 315 nm) are nearly completely absorbed by the DNA and other macromolecules of epidermal cells, i.e. keratinocytes and melanocytes. In contrast, UVA radiation (315 nm – 400 nm) can penetrate deep into our skin and even reach dermal fibroblasts (68, 69). UVA radiation induces oxidative stress and associated macromolecular damage by excitation of endogenous chromophores, such riboflavin and protoporphyrin IX (70). Importantly, the vast majority of skin cancers, i.e. basal cell carcinoma, SCC and malignant melanoma, originate from epidermal cells. Hence, in the context of skin photocarcinogenesis, UVB radiation can be considered as the most dangerous part of the UV spectrum.

### DNA Damage, DNA Repair, and Apoptosis

Skin photocarcinogenesis is a multistep process, involving initiating and promoting events (71, 72). These include DNA damage and failure of appropriate cell rescue (DNA repair) or cell death (apoptosis) responses, the suppression of anti-tumor immune responses, and the clonal expansion of malignant cells (71–74). To provoke effects at a cellular level, UVB radiation needs to be absorbed by chromophores to convert its physical into chemical energy. The most important chromophore for UVB radiation is the DNA (75). In addition, other cellular components, in particular aromatic amino acids, such as tryptophan (76), can absorb UVB photons and contribute to the generation of the UVB stress response in the epidermal compartment (21, 77). The DNA damage-dependent part of this response is initiated by the UVB radiation-induced formation of two photoproducts between adjacent pyrimidine bases: cyclobutene pyrimidine dimers (CPD) and pyrimidine (6–4) pyrimidone photoproducts (75, 78, 79). Although both DNA photoproducts are highly mutagenic, CPDs and the resulting signature mutations, in particular C>T and CC>TT transitions, are considered as being mainly responsible for skin photocarcinogenesis (78, 80, 81). Numerous of these signature mutations are present in the p53 gene, a tumor suppressor gene that is inactivated or compromised by respective mutations in nearly 100% of UV radiation-associated skin cancers (78, 79). In placental mammals, UVB radiation-induced DNA photoproducts (as well as bulky PAH-DNA adducts) are removed by nucleotide excision repair (NER) which consists of four steps: Damage recognition, incision, gap filling and ligation (46, 82). NER is divided into two distinct sub-pathways that differ in their way of damage recognition: Transcription-coupled repair (TCR), which quickly removes DNA adducts in actively transcribed genes, and global genome repair (GGR), which removes DNA lesions in the entire genome. In case of TCR, a stalled RNA polymerase II which then is recognized by Cockayne Syndrome (CS) A and CSB proteins serves as damage sensor. In GGR, a complex of xeroderma pigmentosum (XP) C, centrin-2, RAD23B and DNA damage-binding proteins recognizes the DNA damage. DNA unwinding is performed by the DNA helicases XPB and XPD, which are part of the general transcription factor IIH (TFIIH). Excision of the DNA damage is performed by the endonucleases XPG and XPF-ERCC1 and, subsequently, the gap of 25 to 30 nucleotides is filled and sealed by DNA polymerases and DNA ligases, respectively (46, 82).

In case NER fails, the remaining DNA photoproducts will cause DNA double-strand breaks (DSB), which have been denominated as lethal DNA lesions (83). In fact, DSBs are not a direct consequence of UVB irradiation but occur when CPD-positive cells enter mitosis. During S phase, these helix-distorting DNA lesions cause a collapse of the replication fork leading to breakage (enzymatic cleavage) of both DNA strands (84–86). Subsequently, the DNA damage response, amongst others encompassing the activation of ataxia telangiectasia mutated (ATM) kinase and downstream checkpoint kinase 1 (CHK1), is induced to halt the cell-cycle and initiate homologous recombination repair (HRR) (87, 88), which is primarily in charge of fixing replication fork-associated DSBs (89). When HRR fails, apoptosis is initiated by ATM (or related ATR) kinase in both p53-dependent and p53–independent manners (47). Importantly, an elevation of keratinocyte apoptosis may effectively restrain UVB radiation-induced skin carcinogenesis (90–93).

### UV Radiation-Induced Immunosuppression

As indicated above, UV radiation suppresses the immune system in an antigen-specific manner by inducing Tregs thereby promoting skin carcinogenesis (73, 74). Using mouse models of contact hypersensitivity, the induction of DNA photoproducts, especially CPDs, has been identified to be the major molecular trigger for the UV radiation-induced suppression of the immune system (94, 95). This result has been confirmed in human volunteers treated with UVB light alone or in combination with photolyase-containing liposomes and photo-reactivating light exposure (96). The Tregs induced by UVB irradiation are CD4^+^ and CD25^+^, express FoxP3 and secrete IL-10. Although bearing lymph node-homing receptors, Tregs may switch to skin-homing receptors upon contact with epidermal Langerhans cells, migrate into the skin and suppress cancer cell-killing effector T cells (74, 97). Given that an enforced removal of UVB radiation-induced DNA photoproducts does not completely abrogate immunosuppression (94), other chromophores might also be involved. Besides trans-urocanic acid which upon UVB irradiation may isomerize to immunosuppressive *cis*-urocanic acid in the *stratum corneum* (74), tryptophan is another candidate compound. Indeed, as discussed below in more detail (see *AHR and Immunosuppression*), AHR has been identified to contribute to the suppression of immune responses in UVB-irradiated mice (97) suggesting an involvement of tryptophan photoproducts. FICZ, however, has been reported to enforce the generation of Th17 cells and thereby exacerbate experimental autoimmunity in mice (98, 99).

## Role of AHR in Skin Photocarcinogenesis

### AHR Activation by UVB Irradiation

In the epidermis, UVB rays are absorbed by the aromatic amino acid tryptophan, resulting in the formation of photoproducts, such as FICZ and 1-(1H-indol-3-yl)-9H-pyrido(3,4-*b*)indole, which serve as high-affinity AHR ligands (21, 76, 100–102). FICZ is detectable in human skin *in vivo* (103), and FICZ metabolites, i.e. sulfoconjugates of hydroxylated FICZ molecules, are present in human urine samples. Accordingly, an exposure to UVB radiation enhances cutaneous and hepatic CYP1 enzyme activities in rodents (104, 105), and induces the expression of AHR target genes in the skin of human volunteers (106, 107). FICZ is a very good substrate for CYP1 isoforms and their induction by FICZ-stimulated AHR signaling thus ensures a transient activation of the AHR system in response to acute UVB exposure (108, 109). Even though experimental evidence is lacking, it is tempting to speculate that epidermal AHR activity is also fueled by *N*-formylkynurenine (NFK), another tryptophan photoproduct formed in UVB-irradiated cells (110–112). Subsequently, arylformamidase (kynurenine formamidase) may convert NFK to kynurenine which is further metabolized to endogenous AHR ligands, such as kynurenic acid and xanthurenic acid (113, 114). As discussed later on, this process bypasses the first and rate limiting step of tryptophan catabolism, i.e. the tryptophan 2,3-dioxygenase (TDO)- or indoleamine 2,3-dioxygenase (IDO)-mediated oxidation of tryptophan to NFK, and thus may be relevant for a modulation of UVB radiation-induced immunosuppressive effects by the AHR system. Other genes whose expression is upregulated *via* non-canonical AHR signaling pathways in UVB-irradiated keratinocytes and human skin *ex vivo* and which might be relevant concerning skin carcinogenesis encode for cyclooxygenase-2 and matrix metalloproeinase-1 (21, 106).

Under chronic UVB irradiation, overactivation of AHR signaling pathways may have detrimental consequences (**Figure 3**). In fact, in a photocarcinogenesis study AHR-deficient SKH-1 hairless mice developed approximately 50% less cutaneous SCC than their AHR-proficient littermates, providing evidence that AHR signaling critically contributes to UVB radiation-induced skin carcinogenesis (115). Further analyses of the skin lesions did not reveal any obvious genotype-specific differences in tumor histology/biochemistry (115), indicating that, in the context of photocarcinogenesis, AHR activity mainly affects the tumor initiation phase. However, given that UVB irradiation enhances the expression of inflammatory mediators (e.g. chemokine (C-X-C motif) ligand 5, cyclooxygenase-2) in murine and human skin in an AHR-dependent manner (106, 116), and that a transgenic overexpression of a constitutively active AHR in mice is associated with inflammatory skin lesions (117), it seems to be likely that cutaneous AHR signaling also exhibits tumor-promoting effects.

### AHR and Nucleotide Excision Repair

Given that skin photocarcinogenesis depends on the formation and repair of UVB-induced DNA photoproducts, in particular CPDs, our group has elucidated whether AHR activation affects CPD removal *via* NER. In fact, chemical inhibition and genetic targeting of AHR in human epidermal keratinocytes accelerates CPD removal at early time points (4 hrs) after UVB exposure (115). Treatment of keratinocytes with a pan-caspase inhibitor and subsequent CPD quantification excluded an early clearance of CPD-positive cells through apoptosis. Transient RNAi experiments in which the expression of either XPC, the damage recognition factor of GGR, or CSB, an initiator of TCR, was silenced, revealed that AHR attenuated CPD repair by specifically repressing the GGR sub-pathway (115) (**Figure 3**). The clinical relevance of GGR is illustrated by the fact that patients with GGR-inactivating mutations, but not patients suffering from TCR-deficiency, have a 1,000-fold increased risk to develop cutaneous SCC (118, 119). Further RNAi-based mechanistic studies revealed that AHR inhibits GGR by activating EGFR and downstream PI3K/AKT signal transduction, resulting in the phosphorylation and subsequent proteasomal degradation of the cyclin-dependent kinase (CDK) inhibitor and tumor suppressor protein p27^KIP1^ (115, 120). Accordingly, AHR inhibition results in a stabilization of the p27^KIP1^ protein level in keratinocytes *in vitro* and mouse skin *in vivo* (115, 121, 122). This stabilizing effect of AHR inhibition on p27^KIP1^ is not restricted to epidermal keratinocytes but also present in DAYO medulloblastoma (123) and A549 lung adenocarcinoma cells (124). Ectopic overexpression of p27^KIP1^ accelerates CPD repair in UVB radiation-exposed keratinocytes, whereas chemical inhibitors targeting CDK7, mimicked high levels of p27^KIP1^, i.e. inhibited CPD repair. CDK7 is the catalytically active subunit of the CDK-activating kinase, a component of the general transcription factor and NER complex TFIIH (125). In fact, a chemical inhibition of CDK7 has been previously shown to specifically stimulate GGR activity (126). Elevated p27^KIP1^ protein levels were present in the skin of AHR-deficient mice and associated with a faster removal of UVB radiation-induced CPDs (115). These data indicate that by repressing the repair of UVB radiation-induced DNA photoproducts, AHR may critically contribute to skin photocarcinogenesis. Recently, this concept was challenged by a study reporting that an activation of AHR signaling by keratinocyte growth factor-2 (KGF2) stimulates CPD clearance as early as one hour after UVB exposure (127). However, it is not clear whether this effect depended on alterations in NER activity or apoptosis. Given that peptide growth factors do probably not bind to the ligand-binding site of the AHR protein, the mode of AHR activation by KGF2 remains quite enigmatic. KGF2 serves as a ligand for the fibroblast growth factor receptor-2, which acts mitogenic and thereby may reduce apoptotic cell death (128). Hence, it is tempting to speculate that a cross-talk between the receptor tyrosine kinase and AHR signaling may be causative for the described discrepancy in UVB radiation-induced keratinocyte apoptosis.

### AHR, Homologues Recombination Repair and Apoptosis

As outlined above, an abrogation of AHR signaling accelerates the removal of mutagenic CPDs through the NER sub-pathway GGR and thus should decrease UVB radiation-induced keratinocyte apoptosis (129). However, it has been previously shown that AHR serves an anti-apoptotic function in UVB-irradiated keratinocytes and mouse skin (115, 121). Interestingly, this anti-apoptotic effect also seems to depend on the AHR-mediated reduction of the p27^KIP1^ protein level. Accordingly, an inhibition of AHR enhances the apoptosis susceptibility of UVB-irradiated keratinocytes by upregulating p27^KIP1^ levels (121). Subsequently, p27^KIP1^ inhibits the activity of its substrate CDK2 and thereby abolishes the downstream phosphorylation of the retinoblastoma protein (RB). RB phosphorylation is necessary to activate E2F1, which controls the expression of CHK1 (121), a stress kinase critically involved in initiating cell-cycle arrest upon DNA damage (88). In UVB-irradiated keratinocytes, DNA double-strand breaks (DSBs) mainly occur when CPD-positive cells start to divide (84–86). Results from Comet assays and γH2AX quantification indicated that the enhanced apoptosis susceptibility of AHR-compromised keratinocytes is indeed due to an elevated formation of DSBs (115). Accordingly, at later time points after UVB exposure (18 h), AHR-compromised keratinocytes exhibited an elevated amount of DSBs as compared to respective control cells. Given that CHK1 is also essential for the initiation of HRR (87), AHR-compromised keratinocytes, exhibiting reduced CHK1 levels, are prone to DSB-induced apoptosis (115). These data indicate that AHR is a positive regulator of the HRR (**Figure 3**) and the associated fixation of DSBs and confirm previously published observations in 2,3,7,8-tetrachlorodibenzo-*p*-dioxin (TCDD)-treated Chinese hamster ovary cells (130, 131). However, in contrast to the described anti-apoptotic role of cutaneous AHR in the context of UVB irradiation, it has been reported that in the absence of DNA damage AHR activation may sensitize keratinocytes to cytokine-/death receptor-induced apoptosis (132).

### AHR and Immunosuppression

UVB radiation-induced immunosuppression largely depends on the occurrence of DNA photoproducts (see *UV Radiation-Induced Immunosuppression*). An inhibition of both GGR and apoptotic clearance probably results in an accumulation of CPDs and thus may represent one mechanism through which active AHR maintains the UVB radiation-induced suppression of the immune system. Studies on a mouse model of contact hypersensitivity confirmed this hypothesis by demonstrating that a chemical or genetic inhibition of AHR attenuates the UV radiation-induced expansion of Tregs and associated immunosuppressive effects (97). Further mechanistic studies using 4-n-nonylphenol to induce AHR-dependent immunosuppression, however, showed that AHR activation switches antigen-presenting dendritic cells (DC) from a stimulatory into a regulatory phenotype thereby leading to an induction of Tregs independently from DNA damage (97, 133). The underlying mechanism involves an AHR-dependent induction of IL-2 (133, 134) which subsequently induces IL-10 while repressing the expression of the negative regulatory protein B7-H4, a co-inhibitory molecule of the B7 family (133). In addition, AHR activation by 4-n-nonylphenol induced the expression of IDO in bone marrow-derived DC, thus confirming a previous study reporting that AHR is required for proper IDO induction (135). The enforced degradation of tryptophan and the associated formation of tryptophan metabolites may inhibit T cell proliferation and thereby suppresses antitumor immune responses (114, 136, 137). In fact, the derived tryptophan metabolites may activate AHR to generate regulatory DC which foster the expansion of Tregs while inhibiting T cell polarization towards Th17 cells (114). Noteworthy, kynurenine was found to induce the generation of immunosuppressive Tregs in mice and certain tumor entities in patients in an AHR-dependent manner (136, 138–141). Along the same line, activation of AHR by its prototypic ligand TCDD promotes the expansion of CD4^+^ CD25^+^ and FoxP3^+^ Tregs to suppress experimental autoimmune disease (encephalomyelitis, uveoretinitis) (99, 142, 143). This, however, stands in stark contrast to an activation of AHR signaling by either FICZ (see *UV Radiation-Induced Immunosuppression*) or airborne PAHs and PAH-rich PM (see *AHR and PAH-Induced Immune Reactions*) which stimulate the generation of Th17 cells and exacerbate autoimmune disorders. This discrepancy clearly points to a ligand-specific effect of the AHR system on fate and function of T lymphocytes. Interestingly, results from a study conducted by the Kerkvliet laboratory suggest that this ligand-specific effect is due to differences in the metabolic half-life of the respective AHR ligand and associated dose-dependent effects (144). However, in the context of UVB irradiation, a very interesting facet is that an absorption of UVB rays by tryptophan results in the formation of NFK (110–112), a photochemical reaction which bypasses the first and so-called rate-limiting IDO/TDO-catalyzed step of tryptophan degradation. At biologically relevant doses of UVB radiation and simulated sunlight, approximately 20% of the free tryptophan contained in cell culture medium is converted into the AHR pro-ligand NFK (112). In general, tryptophan is highly susceptible to many oxidizing agents and NFK is one of its major oxidation products (145). Although probably less relevant for the therapy of cutaneous SCC, this observation provides a potential mechanism through which other epidermal cancers, in particular advanced melanomas, may overcome a pharmacological inhibition of IDO/TDO. In fact, a phase III trial (ECHO-301) testing pembrolizumab, an antibody targeting the immune checkpoint protein PD-L1, in combination with the IDO blocker epacadostat revealed that the co-treatment has no benefit for patients suffering from advanced melanoma as compared to the patients treated with pembrolizumab alone (114, 146). The failure of IDO1 inhibitors for melanoma therapy might be related to an enhanced TDO activity or an elevated expression of IL-4-induced gene 1, another tryptophan-metabolizing enzyme that induces immunosuppression by producing AHR ligands (147, 148). Nevertheless, it is tempting to speculate that the UV radiation-induced formation of NFK in the skin and its further catabolism to kynurenic acid and other AHR-agonistic metabolites may, at least partially, contribute to the expansion of Tregs and the suppression of appropriate anti-tumor immune responses. As indicated by studies on patients with lung cancer or oral SCC, the activated AHR may not only enhance the expression of IDO but also attenuate the response to immune checkpoint inhibition by inducing PD-L1 (141, 149). In addition, tumor repopulating melanoma cells may produce kynurenine and associated AHR ligands to activate AHR and induce the expression of the PD-L1 receptor PD-1 in CD8^+^ T cells (150). Hence, a combination of small molecules inhibiting AHR activity with PD-1 checkpoint inhibitors might be a suitable approach to combat immunotherapy-resistant tumors (151).

Although experimental evidence is yet lacking, it is conceivable that at least in advanced stages of cutaneous SCC, the AHR may play a comparable role in modulating tumor immunity.

## Co-Carcinogenicity of UV Radiation and PAHs?

Given that environmentally ubiquitous PAHs as well as UV radiation are capable of modulating AHR activity in keratinocytes and other epidermal cell populations, a simultaneous exposure to these factors may cause co-carcinogenic effects. However, as specified below, the data published so far on this topic produce a heterogenous and sometimes even contradictory picture. For instance, some early carcinogenesis studies reported an increased skin tumor formation upon UV irradiation of coal tar-treated mice (152, 153), whereas in another study on albino mice alternately exposed to UV radiation and PAHs, namely 3-methylcholanthrene, DMBA and B[a]P, no additive skin cancer formation was observed (154). A major factor that may contribute to this discrepancy is the pronounced sensitizing property of various PAHs toward UVA radiation. The resulting generation of ROS may on the one hand facilitate carcinogenesis by inducing oxidative DNA damage and inhibiting the function of DNA repair enzymes and other proteins (155–162). On the other hand, strong or longer lasting phototoxic stress may cause ROS-mediated cytotoxicity and necrotic cell death not only in normal epidermal cells but also in initiated keratinocytes and malignant cells. Interestingly, several UVB radiation-induced photoproducts of tryptophan, including NFK and FICZ, have been identified as potent UVA photosensitizers (110, 163, 164), and a combinatorial treatment with FICZ and UVA radiation was proposed as a novel therapeutic approach for skin cancer (165). Notably, other investigators regard the oxidative stress resulting from the FICZ/UVA exposure and the associated inhibition of DNA repair enzymes as a pro-carcinogenic event (166). In general, this process, i.e. the application of a photosensitizing agent and its subsequent irradiation with light of a certain wavelength, has been successfully implemented into the clinical routine for the treatment of certain solid tumors (167), including melanoma and non-melanoma skin cancers (168), and is known as photodynamic therapy. Other studies have shown that UV irradiation enhances the skin permeation rates of simultaneously applied PAHs (161, 169, 170) and may affect their metabolic activation (105, 155, 171, 172). UVB radiation was proven to sensitize epidermal keratinocytes to PAH-DNA adduct formation and subsequent mutagenesis (105, 171). The laboratory of David Bickers applied the Goeckerman regimen, i.e. a sequential treatment of the skin with PAH-rich crude coal tar and UVB radiation, to neonatal rats and observed an enhanced metabolic activation of B[a]P and associated DNA adduct formation in subsequent *ex vivo* experiments (105). An enhanced amount of PAH metabolites and markers for DNA damage were also observed in the blood and urine of psoriasis patients that underwent the Goeckerman regimen (161). Interestingly, Bickers and co-workers were able to show that the opposite application sequence, i.e. UVB exposure first followed by coal tar treatment, did not cause any significant differences in metabolic activation and BPDE-DNA adduct formation as compared to the samples of the coal tar-only treated animals (105). In contrast, Nair and colleagues reported that a treatment of HaCaT keratinocytes with either UVB radiation, photooxidized tryptophan or FICZ prior to B[a]P application significantly enhanced the expression of CYP1 isoforms and the associated formation of bulky DNA adducts (171). As expected a co-treatment with either α-naphthoflavone, an AHR antagonist, or the HSP90 blocker 17-AAG attenuated CYP1 induction and DNA adduct formation, thus confirming AHR dependency (171). Thierry Douki and co-workers, however, reported that a sequential treatment of keratinocytes and human skin explants with B[a]P or a mixture of PAHs and stimulated sunlight, reduced the expression of CYP1 isoforms, the generation of PAH metabolites and BPDE-DNA adduct formation (155, 170, 172). Given that the applied irradiation device emits UVB and UVA light (wavelengths from 290 nm - 400 nm), it is possible that the resulting generation of ROS is responsible for the observed downregulation of the expression of the CYP1 monooxygenases (173, 174). In addition, the spectrum of cytokines released by the irradiated epidermal cells may depend on the UV wavelength (175, 176). Tumor necrosis factor-α, for instance, is rather produced upon irradiation with UVA than with UVB light, and this cytokine is capable to suppress CYP1 gene expression *via* NF-κB-mediated trans-repression, i.e. a competition for common transcriptional co-activators (24, 177). However, as outlined in this article, PAHs and UV radiation may differently affect the expansion and function of immunosuppressive Tregs, which may be also an important factor that has to be considered in the context of a simultaneous exposure.

In summary, the current data concerning the simultaneous exposure of the skin to UV radiation and PAHs are not coherent and illustrate the need for further studies to ensure a proper risk assessment, in particular for roofers, roadmen, and other occupational groups that are frequently exposed to high doses of both, PAHs and sunlight. Chronic exposure studies on rodents, for instance, using the same irradiation device for single and simultaneous exposure to UVA and UVB light in combination with a pre- and post-treatment with environmentally-relevant PAH mixtures may shed light on a potential interaction of both risk factors in skin carcinogenesis. 

## Conclusion

Proper AHR signaling is indispensable for the development and physiology of the skin (178, 179). However, as outlined in this article, an overactivation of this signaling pathway in skin chronically exposed to one or more environmental stressors may have detrimental consequences (**Figure 3**). By modulating xenobiotic metabolism, different DNA repair systems, apoptosis, various functions of the immune system, and other processes, AHR-dependent signaling pathways may significantly contribute to the development of PAH- and UV radiation-induced skin carcinogenesis. Interestingly, both environmental factors seem to interact on the level of enzyme activity and DNA damage and repair, thus illustrating the critical role of the AHR in either restraining or facilitating the development of skin cancer. Even though, the published data on the potential co-carcinogenic action of UV radiation and PAHs do not produce a clear picture, a transient inhibition of cutaneous AHR signaling probably protects the skin of individuals exposed to UV radiation and PAHs alone or in combination. In the context of a co-exposure, it is tempting to speculate that the application of sunscreen does not only prevent the UV radiation-induced activation of AHR signaling but also the photoactivation of PAHs potentially present on the skin. Given that sun blockers do not protect against the genotoxicity of PAHs, the integration of transient AHR antagonists in sunscreens might be beneficial in order to protect the skin against both environmental/occupational stressors. Notably, it is widely accepted that the integration of antioxidants into sunscreens provides additional skin protection by neutralizing radiation- and pollution-induced ROS (180, 181) and, interestingly, several plant-derived polyphenols combine both properties, i.e. antagonizing AHR signaling and exhibiting antioxidative effects (182, 183). However, further research on the potential interaction of PAHs and UV radiation in the pathogenesis of SCCs is urgently needed in order to improve the risk assessment as well as the preventive strategies depending thereon.

## Author Contributions

TH-S contributed to the conception and design of the review article. CV, KR, and TH-S wrote the manuscript. JK provided critical feedback during the preparation of the article and contributed to the revision of the manuscript. CV generated the figures. All authors contributed to the article and approved the submitted version.

## Conflict of Interest

The authors declare that the research was conducted in the absence of any commercial or financial relationships that could be construed as a potential conflict of interest.

## Publisher’s Note

All claims expressed in this article are solely those of the authors and do not necessarily represent those of their affiliated organizations, or those of the publisher, the editors and the reviewers. Any product that may be evaluated in this article, or claim that may be made by its manufacturer, is not guaranteed or endorsed by the publisher.
